# Metabolomics, Oxidative, and Nitrosative Stress in the Perinatal Period

**DOI:** 10.3390/antiox11071357

**Published:** 2022-07-12

**Authors:** Julia Kuligowski, Máximo Vento

**Affiliations:** 1 Neonatal Research Group, Health Research Institute La Fe (IISLaFe), 46026 Valencia, Spain; maximo.vento@uv.es; 2 Division of Neonatology, University and Polytechnic Hospital La Fe (HULAFE), 46026 Valencia, Spain

The perinatal period is extremely sensitive to external stimuli, and events that may disturb the equilibrium within the mother–infant dyad might have a substantial short- and long-term impact on the infant’s health and development. Lacking oxygen in the perinatal period can result in bioenergetic failure and cell death, with consequences for infant health and survival. While the administration of supplemental concentrations of oxygen can be lifesaving, it can also disrupt growth and development, impair bioenergetic function, and induce inflammation. Oxidative stress is of key importance in the pathophysiology of several diseases, affecting term as well as preterm infants. Professor OD Saugstad referred to these effects as the “Oxygen radical diseases of Neonatology” [1]. This important topic is addressed in this Special Issue in a review article which describes the historical perspective and current trends in the use of supplemental oxygen in newborns, covering unique features of newborn redox physiology and antioxidant defenses, the history of therapeutic oxygen use in this population and its role in disease, clinical trends in the use of therapeutic oxygen and mitigation of neonatal oxidative injury [2].

Sex differences in the susceptibility to oxidative-stress-related complications of prematurity are a widely accepted concept. The foundation of this theory is elucidated in a systematic review and meta-analysis of cohort studies exploring the association between sex and complications of prematurity, showing that male preterm male infants have higher clinical instability and greater need for invasive interventions than female preterm infants [3]. This leads to a male disadvantage in mortality and explains differences in short-term complications of prematurity.

Human milk is a globally recognized gold standard for infant nutrition, and is especially important for the healthy growth and development of preterm infants. Furthermore, the antioxidant properties of human milk mitigate the consequences of excessive oxidative damage. When the mother’s own milk is unavailable, pasteurized donor milk is the best alternative. Although pasteurization is necessary for safety reasons, it may affect the concentration and activity of biological factors, including antioxidants. This Special Issue includes a review article describing the effect of different pasteurization methods on antioxidant properties of human milk that aims to provide evidence to guide donor milk banks in choosing the best pasteurization method from an antioxidant perspective [4].

In recent years, and with the advent of powerful high resolution and high-throughput analytical methods, researchers have begun to successfully develop and implement novel metabolomics approaches for diagnostics in perinatal asphyxia. It is important to design non-invasive tools tailored to the characteristics of newborns, and saliva analysis has unexplored potential. This Special Issue includes a literature review providing an overview of metabolomics studies of oxidative stress in perinatal asphyxia, particularly seeking studies analyzing non-invasively collected biofluids such as saliva [5]. While changes in oxidative stress-related salivary metabolites have been reported in adults, the utility of this approach in perinatal asphyxia has yet to be explored. Experimental and clinical studies indicate that, in addition to antioxidant enzymes, succinate and hypoxanthine, acylcarnitines may also have discriminatory diagnostic and prognostic properties in perinatal asphyxia. Accumulating evidence of discriminatory metabolic patterns in perinatal asphyxia may be useful to develop point-of-care methods for measuring salivary oxidative stress metabolites relevant in perinatal asphyxia.

The versatility of metabolomics and the use of this methodology for a sensitive detection of transient changes in longitudinal studies has been demonstrated in a study involving newborns with transposition of the great arteries (TGA), a common cyanotic congenital heart disease [6]. Parallel circulations that result in impaired cerebral oxygen delivery in utero may lead to brain damage and long-term neurodevelopmental delay. To mix deoxygenated and oxygenated blood at the atrial level, balloon atrial septostomy (BAS) is frequently employed, causing a sudden increase in arterial blood oxygenation and oxidative stress. Changes in oxygen saturation as well as metabolic profiles of plasma samples from nine newborn infants suffering from TGA before and until 48 h after undergoing BAS were recorded. The plasma metabolome changed clearly over time, and alterations of four metabolic pathways, including the pentose phosphate pathway, were linked to changes in the cerebral tissue oxygen extraction. On the contrary, no changes in levels of lipid peroxidation biomarkers were observed. These findings suggest that metabolic adaptations buffer the free radical burst triggered by re-oxygenation, thereby avoiding structural damage at the macromolecular level.

Neonatal encephalopathy is one of the main causes of morbidity and mortality in term infants, and there is evidence that oxidative damage plays an important role in the pathophysiology of hypoxic–ischemic brain injury. Recently, several studies have been conducted with the aim of providing adjacent neuroprotective and antioxidant therapeutic options complementary to hypothermia treatment, animal [7,8,9] and clinical [10] studies with different compounds. Kynurenic acid significantly reduced reactive oxygen species and antioxidant enzyme activity and enhanced GSH levels and hypoxic–ischemic conditions in a rat model [7]. Only the highest concentration of kynurenic acid showed neuroprotection when applied 6 h after hypoxia–ischemia, and results indicated the induction of neuroprotection at the reactive oxygen species formation stage. In a different study, the capability of postnatal allopurinol administration in combination with hypothermia treatment to reduce oxidative stress biomarkers was assessed in a rat model of hypoxic–ischemic encephalopathy, showing that the administration of allopurinol, hypothermia, and the combination treatment protects the brain against oxidative damage [8]. Furthermore, in neonatal hypoxia ischemia complicated by perinatal infection, therapeutic hypothermia does not improve outcomes due to pre-existing oxidative stress and neuroinflammation, which shorten the therapeutic window. Hence, the definition and targeting of central nervous system metabolomic changes immediately after endotoxin-sensitized hypoxia–ischemia (LPS-HI) has been studied in a rat model with the aim of discovering neuroprotection strategies that are effective post-injury [9]. Despite hypothermia treatment, LPS-HI acutely depleted reduced glutathione, indicating overwhelming oxidative stress. However, the combination of the administration of N-acetylcysteine and vitamin D (NVD) with hypothermia rapidly improved cellular redox status after LPS-HI, potentially through the inhibition of important secondary injury cascades, allowing more time for hypothermic neuroprotection. The promising results obtained in this experimental study were further validated in a clinical trial that aimed to translate these FDA-approved drugs to HIE neonates [10]. NVD was well tolerated and 24 treated HIE infants had no evidence of cerebral palsy, autism, or cognitive delay at 24–48 months, confirming that low, safe doses of NVD in HIE neonates decrease oxidative stress, improve central nervous system energetics, and are associated with favorable long-term developmental outcomes.

In summary, this Special Issue demonstrates the potential of the use of metabolomics and the assessment of oxidative stress for gaining insight in a variety of relevant aspects related to the health and disease of newborns, as well as new therapeutic approximations.
